# Nitrate of Lead as a Disinfecting Agent

**Published:** 1849-01

**Authors:** 


*Nitrate of Lead as a Disinfecting Agent.*—Nitrate of lead has been employed recently in the Com. Hospital of this city, as a disinfecting agent, and the experiments thus far made, promise the most satisfactory results. The power of this article to correct the odor arising from decomposing morbid specimens, was accidentally noticed by Dr. Cary, resident physician at the Commercial Hospital; and taking advantage of this discovery, he employed it for the purpose of removing the odor of alvine discharges, offensive ptyalism, &c., with the most complete success. It entirely removes the odor of decomposing animal substances, alvine excretions, sloughing or offensive ulcers, and may doubtless be made a valuable agent in the treatment of any disease requiring such an agent. It is entirely soluble in water, and possesses astringent properties, similar to the acetate of lead.

It may be used in the proportion of thirty to one hundred grains to the ounce of water. Dr. Cary is still experimenting with the agent, and promises to report the results for publication in the *Lancet.*

Professor Mussey has frequently used nitrate of lead, as an application to offensive ulcers.—*Western Lancet.*

